# Animal Heat.—an Inaugural Thesis

**Published:** 1867-04

**Authors:** W. A. Grahame


### ANIMAL HEAT.—AN INAUGURAL THESIS.

BY W. A. GRAHAME, D. D. S.

In offering a thesis upon a subject like this, we presume that it is not expected that anything new will be advanced, because it seems that nearly all has been said that can be, unless scientific research should, hereafter, develop some things that are now unknown. Then, it only falls within our province to say what we know and have read on the subject.

Heat, in all its modifications, is a very abstruse subject. It is an agent that man will use in almost numberless ways, and never give himself over to thinking of its mysterious qualities. "The unlettered rustic" will put some wood on the fire, sit down and warm himself by it, and never wonder *why* the fire burns, nor *how*. The blacksmith puts his iron into the fire, not knowing *why* the heat destroys the cohesiveness of its particles, so that they are made movable among each other; nor does he care, so long as it serves his purposes. And indeed, we all use heat for mechanical and other purposes, not knowing the amount required for the one thing, nor the reason for the other.

But, in entering into a short essay on the subject of VITAL HEAT, we will define (as nearly as possible), *heat* to be the sensation produced when we approach near a warm body; and *caloric* is said to be the *cause* of that sensation.

*Animal heat* is that heat existing in all warm-blooded animals, which is necessary to their existence as living beings; but cold-blooded animals, too, have a certain amount of this heat, although so little that it can scarce be noticed to be above that of the element in which they live, which may be accounted for on the ground that they have feeble circulations, and their food is of that kind that does not produce much heat; but it is presumed that it is produced in all those animals that give off carbonic acid in respiration. The temperature of the human body is about 98° Fah., but varies a little according to circumstances. As an instance, exercise, active, vigorous exercise, causes an accelerated circulation, an increase of food is thereby demanded, more oxygen is taken into the system; consequently, more heat will be generated. Then, on the contrary, rest or sleep will have an opposite effect, because respiration is somewhat diminished, less carbonic acid is thrown off, and the temperature necessarily falls a little—perhaps as much, or nearly so, as it was increased by the exercise. Age, diseases, habits, &c, have some influence, and cause a slight variation of the temperature of the body. A man thinly clothed on a wintry day will wish for more food to supply the deficient amount of Animal Heat, because nature demands that the temperature remain at nearly the same degree at all times. In the febrile diseases, the amount of heat is sometimes very greatly increased. Cases of typhoid fever sometimes reach 103° and 104° Fah., and even higher. One case of tetanus rose to 110¾. This high temperature often falls very rapidly, and with it a corresponding retardation of the pulse; but in such diseases as cholera or yellow fever, quite the reverse will be observed. In cholera, when the respiration becomes difficult, and the patient changes from a natural to a dark color, we suppose the blood is being deprived of its due amount of oxygen. Then the temperature falls very low, according to some observers as low as 74° Fah. So we cannot but believe that respiration and Animal Heat are very closely connected.

We do not know that the ancients were in posession of any very well defined theories on the subject of Vital Heat. Many of their best physiologists believed that it was generated in the lungs and heart. Some thought in the lungs alone, and others in the heart. Mayo believed and taught that the *object* of respiration alone was to produce heat, and refuted the doctrine that the blood was cooled in the lungs, which was taught by some at that day. Black's theory was that breathing caused a combustion in the lungs which produced heat. His was not very generally received, because if the lungs generated all the heat, it was thought that, like a furnace, they and the parts immediately about them would be much warmer than the more remote parts of the system. Dr. Crawford advanced this theory, that Animal heat was generated in the lungs, and that arterial blood had a greater capacity for heat than venous ; but as an argument against this theory, Dr. Davy, near that time, demonstrated the capacity for heat in both kinds of blood to be nearly equal, and the result was that his theory was generally abandoned. Sir Benjamin Brodie, Chossat, and some others maintained that Animal Heat was dependent upon and produced by the action of the brain, nerve centers and nerves, they being active, a change of particles takes place, and thus produces this heat. And, indeed, it cannot be doubted that the nervous system has its share of the influence on the heat of the body, as proof of which, the advocates of this theory argue that a paralyzed limb or portion of the body is always colder than if possessing its normal nervous power; or an animal, with the sympathetic nerve divided has an increase of heat, and if under the influence of chloroform, the temperature will fall, and rise again when the influence has passed away. The brain and nerves seem to have some effect on the heat of an intoxicated man, that he, stimulated by liquor, can subject himself to so great a degree of cold and not apparently suffer, as he otherwise would not be able to do. However this may be, owing partly to another cause. Professor Sontag, astronomer to Dr. Kane's expedition to the arctic regions, in his history of that expedition, speaks of the inhabitants of those regions keeping up Vital Heat in a great measure by eating meat and drinking warm seal oil ; and such is the case among the Esquimeaux and Greenlanders; and Dr. Kane's men had not long been in those cold countries until they were eating and drinking great quantities of the same, and found that they were thereby enabled to endure a much greater degree of cold. The climate seemed to create the appetite for that kind of food which produced the greatest amount of heat, or that contained the greatest amount of carbon and hydrogen, which enter into the tissues, uniting with the oxygen, and thus producing heat. The case is reversed with those in the tropical climates; for we find them subsisting almost entirely on fruits, vegetables, &c., food that is not calorifacient. But the theory that has received the sanction of most physiologists of this day, is that Animal Heat is developed on the production of carbonic acid by the combustion of carbon and the consumption of oxygen, which is accomplished in several ways, as in food, respiration &c., although it does not appear that it is altogether dependent on this and nothing else, but no doubt it is in a great measure so. The inference may be drawn that it is developed by means of chemical changes that are always taking place while life lasts, each organ performing its duty in the matter. That heat is developed by oxygen uniting with other substances, is self-evident, and the process of oxydation gives off as much heat when it goes on slowly as when fast. "An iron rod burned in oxygen gas gives off no more heat than if merely oxydized in open air," but it is much more perceptible. The heat of the body is general, but may be, as by local inflammation, locally increased; that is, in the inflamed part, and yet the principal part of the blood will remain at its normal degree. But an increase of heat in the body, or general system, must be caused by a greater action of the organs producing this heat, and a decrease, the want of this action. It has been said that Vital Heat and respiration are closely connected. An objection brought against this theory by some, is, that if oxygen taken into the lungs produces heat, that nature would have made the proportions of oxygen to those of nitrogen in atmospheric air greater than really *now* exist. The reason nature has not done this is that it would have been an extreme equally as injurious as too little oxygen would be, which is demonstrated clearly by inspiring nitrous oxyd gas, the effects of which are well known. In regard to the Vital Heat of animals, birds, fishes, &c., &c., much might be said of interest. In a few words, however, it is presumed that it may be accounted for on these same physiological facts and principles that have just been mentioned. But we see a great difference. Those animals which hibernate lose many degrees of heat, because of inaction and loss of fat.

We observe the temperature of birds to be near 104° Fah. They have active circulation of the blood, and respiratory organs larger in proportion than man. Fishes that breathe by means of lungs have a temperature of nearly 45° above those that breathe by means of gills. So we see that heat is a very common result of chemical action; but there is something difficult to understand in its production, distribution and assimilation. The food of plants is taken from the earth, air and water, and by means of their organs is appropriated to the nourishment and growth of the same, forming the various compounds of the plants which make the food, fuel, &c., for the animal kingdom; and, again, by the life, death and decay of which these agents are set free, and go to the formation of others.

Thus it is that we live. In all the processes and the economy of life, there is a system, and these are mutually dependent on each other; and that this system is as truly founded on laws, fixed, we are as sure as we are, that astronomical calculations are based upon the fixed mathematical laws of geometry, or the measurement of straight lines and circles. But yet we are in ignorance of many of these laws; indeed, we think they are only known to the great God who made man "and all things else," and breathed into him the breath of life, and he became a living, *warm*, breathing soul.

